# Cortical evoked responses to evaluate the effect of spinal cord stimulation on the pain pathways

**DOI:** 10.1016/j.cnp.2025.04.003

**Published:** 2025-05-03

**Authors:** Laurien J. Reinders, Cecile C. de Vos

**Affiliations:** Erasmus University Medical Centre, Centre for Pain Medicine, Anaesthesiology, Dr. Molewaterplein 40, 3015 GD Rotterdam, the Netherlands

**Keywords:** Chronic pain, Conditioned pain modulation, Neurostimulation, Spinal cord stimulation, Cortical activity, Somatosensory evoked response, Magnetoencephalography

## Abstract

**Objectives:**

The mechanisms of spinal cord stimulation (SCS) are insufficiently understood. Conditioned pain modulation (CPM) measures how a painful stimulus is affected by a second painful stimulus. We investigated whether cortical evoked response can be used to evaluate CPM in a patient treated with burst, tonic and sham SCS.

**Methods:**

A 40-year-old patient underwent 3 magnetoencephalography sessions (burst, tonic, sham SCS) with 1-week intervals. Painful electrical stimuli were applied to the tibial nerve before, during and after CPM (conditioning: icepack on forearm). Evoked responses were analysed in the primary somatosensory and anterior cingulate cortices.

**Results:**

Before CPM, the highest evoked response amplitude occurred under sham SCS, followed by tonic SCS. During CPM pain ratings remained unchanged. However, CPM reduced evoked response amplitudes in the primary somatosensory cortex under tonic and sham SCS and in the anterior cingulate cortex under all SCS paradigms.

**Conclusions:**

CPM reduced evoked response amplitudes, while pain ratings were unaffected, suggesting neurophysiological measures provide additional insights into CPM effects. Tonic and burst SCS both appeared to reduce cortical capacity to attend to stimuli, with burst showing the greatest effect.

**Significance:**

Cortical responses offer a valuable tool to assess pain pathways. Larger scale studies are needed to enhance our understanding of SCS mechanisms.

## 1 Introduction

### 1.1 Chronic pain

Chronic pain, defined as pain that recurs or persists for more than 3 months (Treede et al., 2019), has a high and increasing prevalence and is associated with a high disease and disability burden globally (Ferreira et al., 2023). Despite the significant impact of chronic pain on individuals and society, it remains a challenging condition to understand and treat effectively due to its complex and multidimensional nature. The subjective nature of (chronic) pain poses challenges in accurately quantifying and characterizing it, as assessments often rely on subjective self-reported pain ratings.

### 1.2 Conditioned pain modulation (CPM)

Nociceptive inputs can be inhibited or enhanced by endogenous pain modulation via the descending pain pathway. Conditioned pain modulation (CPM) is a centrally processed measure of the net effect of the descending pain pathway (Ramaswamy and Wodehouse, 2021). CPM relies on the ‘pain inhibits pain’ theory, where a first painful stimulus (test stimulus) is inhibited by applying a second painful stimulus (conditioning stimulus) (Ramaswamy and Wodehouse, 2021). The phenomenon was first described in 1979 in rats (Le Bars et al., 1979) and termed diffuse noxious inhibitory control and attributed to the spino-bulbo-spinal loop. In this loop the conditioning stimulus produces nociceptive signals ascending to the brainstem, which triggers descending modulation of wide dynamic range neurons in the spinal dorsal horn, ultimately inhibiting the test stimulus. To standardize terminology, it was recommended by a panel of experts to use the term CPM to describe the observed pain inhibitory effect in humans (Yarnitsky et al., 2010). CPM relies on the spino-bulbo-spinal loop and as well involves psychophysical components (Ramaswamy and Wodehouse, 2021).

Endogenous pain modulation is often disturbed in patients with chronic pain, observed as a less efficient CPM (Ramaswamy and Wodehouse, 2021). In a previous study (Jin et al., 2023) we observed a reduced inhibitory effect by CPM on the cortical response to test stimuli in chronic pain patients, suggesting a disrupted descending pain pathway. While cortical responses showed a clear reduction during CPM, the decrease in subjective pain ratings of the test stimuli was minimal (0.5/10 in healthy controls vs. 0.2/10 in chronic pain patients). These findings indicate that cortical responses may provide additional insights into CPM, complementing subjective pain ratings and offering a broader perspective on pain processing. We are now extending this research to explore the effects of various spinal cord stimulation (SCS) paradigms on the pain pathways.

### 1.3 Spinal cord stimulation (SCS)

Spinal cord stimulation (SCS) is an implantable neurostimulation technique for patients with severe, otherwise intractable chronic neuropathic pain. SCS is a last resort treatment and has a long-term success rate of 57 to 81 %, depending on the aetiology (Cameron, 2004). Despite the relatively high success rate, a group of patients with chronic pain who do not benefit from SCS remains. To understand the reasons why SCS is not effective in all patients with chronic pain, it is crucial to further unravel the mechanisms of action of SCS. The mechanisms of SCS are most likely based on changing the neural activity in several spinal and supraspinal areas, however they are still insufficiently understood (Caylor et al., 2019). One proposed mechanism of SCS is its influence on the endogenous pain modulation through its effect on the descending pain pathway (De Ridder and Vanneste, 2016).

The conventional SCS paradigm comprises tonic pulses applied to the dorsal column, with the goal of inducing comfortable paraesthesia (tingling sensation) in the area innervated by the stimulated nerve fibres. Over the last decade paraesthesia-free SCS paradigms have been developed, such as burst SCS. It has been suggested that burst SCS may act through different mechanisms of action than tonic SCS (De Ridder and Vanneste, Jan 2016, Niso et al., 2021). CPM offers a valuable approach to evaluate the effects of the various SCS paradigms on the pain pathways.

### 1.4 Magnetoencephalography (MEG)

We used magnetoencephalography (MEG) to measure the cortical response to evaluate CPM, moving beyond self-reported pain ratings. This approach provides insights into the activity of cortical structures related to the processing of painful somatosensory stimuli and reflects spinal and supraspinal mechanisms of SCS and CPM. MEG offers an excellent temporal resolution, with still a relatively good spatial resolution, of the cortical responses.

### 1.5 Aim

This case study aims to assess whether the cortical evoked response can be used to evaluate the effect of conditioned pain modulation (CPM) in a patient treated with burst, tonic and sham spinal cord stimulation (SCS) paradigms. Our ultimate goal is to understand the effect of SCS on the pain pathways.

## 2 Methods

### 2.1 Case presentation

A 40-year-old female with persistent spinal pain syndrome type 2 for 5 years was implanted with spinal cord stimulation (SCS) 2 years ago. She has chronic pain in her right lower leg and foot. The patient is satisfied with SCS treatment and uses a tonic stimulation paradigm. Additionally, she takes tramadol and paracetamol.

### 2.2 Magnetoencephalography measurements

Magnetoencephalography (MEG) measurements were performed at the Donders Institute for Brain, Cognition and Behaviour (Nijmegen, the Netherlands). Data were acquired with a whole-head MEG system (275-axial gradiometer channels: CTF, Coquitlam, BC, Canada) in a passive magnetically shielded room. Ethics approval was obtained from the Institutional Review Board. The patient provided written informed consent.

The patient underwent 3 MEG sessions, with 1-week intervals. In the week prior to each session, the patient had 1 of the 3 stimulation paradigms. The first session the patient had a burst paradigm (40 Hz, 1000 μsec, 5 spikes 500 Hz, 0.6 mA), the second session a tonic paradigm (30 Hz, 200 μsec, 1.5 mA) and the third session a non-therapeutic sham paradigm (40 Hz, 100 μsec, 2 spikes 500 Hz, 0.05 mA).

Each session, MEG measurements were made to record the evoked responses elicited by the test stimuli during 3 conditioned pain modulation (CPM) conditions: before, during and after CPM. A 2-min interval separated each CPM condition. This design allowed us to assess CPM using both a parallel paradigm (during CPM condition) and a sequential paradigm (after CPM condition), which are both used in the standard clinical CPM testing (Ramaswamy and Wodehouse, 2021).

Each CPM condition included application of 22 test stimuli. Test stimuli consisted of painful transcutaneous electrical stimuli applied at the right tibial nerve with Ag-AgCl electrodes placed at a distance of 2.5 cm, the stimuli were generated by a constant current stimulator (DS7A, Digitimer Ltd). Test stimuli were applied at randomized interstimulus intervals of 6 to 10 sec to minimize stimulus predictability. Before the measurements, we presented a short series of stimuli with ascending and descending intensity to identify the intensity that induced a pain rating of 5 on a 0 to 10 numeric rating scale.

The test stimulus was accompanied by a conditioning stimulus during CPM, consisting of an ice pack on the left forearm. Although a cold water bath is more commonly used as conditioning stimulus, this was not feasible in combination with the MEG scanner, using an ice pack as conditioning stimulus was shown to be an effective conditioning stimulus in previous EEG and MEG studies (Jin et al., 2023, Ladouceur et al., 2018, Rustamov et al., 2020). Directly after each CPM condition, the patient rated the average pain intensity of the 22 test stimuli (and conditioning stimulus) on a 0–10 numeric rating scale. The full CPM protocol is described by Jin et al. (2023).

### 2.3 Data analysis

Data analysis was performed with Brainstorm (version 3 2022) (Tadel et al., 2011), which is documented and freely available for download online under the GNU general public license (https://neuroimage.usc.edu/brainstorm).

Pre-processing of the raw MEG data included: removal of stimulation artifact (0–30 ms post-stimulus), eye blinks and cardiac activity; application of a bandpass filter (1–200 Hz) and notch filters for powerline contamination. A head model was generated using an overlapping spheres approach. We used the default MRI ICBM125 and warped the MRI using digitized head points. Source estimation was performed using a minimum norm imaging approach with unconstrained sources (Brainstorm).

### 2.4 Regions of interest

2 regions of interest were defined: the foot area of the left primary somatosensory cortex (S1) and anterior cingulate cortex (ACC). The average evoked response elicited by the test stimuli was extracted for both regions of interest for each CPM condition and SCS paradigm and was z-scored with respect to baseline [-2 to −0.5 s]. The area under the curve was calculated from 40 ms till the point at which the final activity was observed under any of the paradigms. The percentage change of area under the curve was calculated between the CPM conditions.

The vertex is often used as region of interest in EEG studies studying CPM and includes the primary somatosensory cortices associated with perceiving pain (Moont et al., 2012). During CPM we expect a decreased amplitude of the evoked response at the left S1 related to the area of the foot where the test stimulus is applied, as previous EEG studies observed a decreased amplitude at the vertex in healthy individuals (Brock et al., 2012, Moont et al., 2012), reflecting reduced pain perception during CPM.

The ACC is thought to contribute to emotional aspects of pain and descending pain inhibition (Brock et al., 2012). Previous EEG studies in healthy individuals showed increased activity in frontal cortical areas during CPM up to 450 ms post-stimulus (Brock et al., 2012, Moont et al., 2012), likely attributed to increased ACC activity (Brock et al., 2012). Additionally, Moont et al. (2012) reported decreased ACC activity 450–600 ms post-stimulus. Therefore, we expect that in the ACC the amplitude of the evoked response of earlier components increases and of later components decreases.

## 3 Results

### 3.1 Pain ratings

The patient rated her average ongoing lower leg and foot pain 1/10 under tonic SCS, 4/10 under burst SCS and 5/10 under sham SCS (Table 1). She reported no difference in pain rating of test stimuli between the before and during conditioned pain modulation (CPM) conditions under all SCS paradigms (Table 1).

**Table 1:** Reported pain ratings for the patient’s ongoing own chronic pain, test stimulus and conditioning stimulus.

**SCS paradigm**	**Pain rating**				
	**Patient’s own pain**[NRS 0–10]	**Test stimulus**			**Conditioning stimulus**[NRS 0–10]
		Before CPM [NRS 0–10]	During CPM [NRS 0–10]	After CPM [NRS 0–10]	
Tonic	1	2	2	2	4
Burst	4	2	2	3.5	5
Sham	5	4	4	4.5	6

*Pain ratings are provided on a NRS from 0 to 10. Pain ratings of test stimuli were providedafter each CPM condition (average rating of 22 test stimuli) in a patient receiving burst, tonicand sham SCS paradigms*.

*CPM = conditioned pain modulation; NRS = numeric rating scale; SCS = spinal cord stimulation*.

### 3.2 Effect of CPM on cortical evoked response

**Fig. 1:**
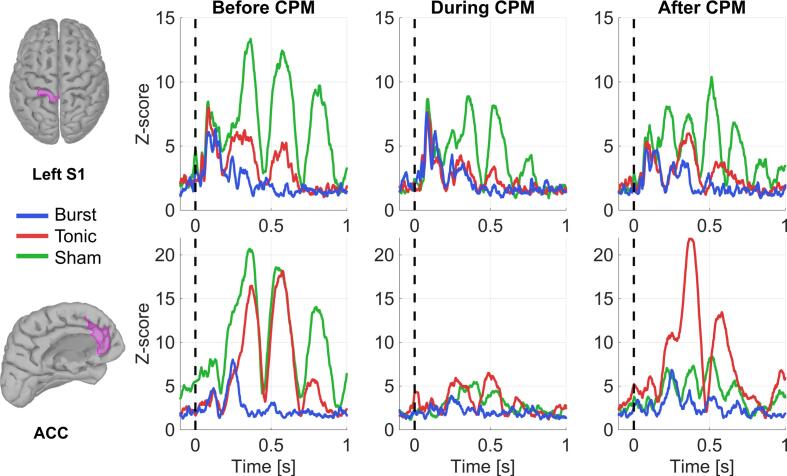
The averaged evoked responses in the foot area of the left primary somatosensory cortex (S1) and anterior cingulate cortex (ACC) elicited by painful electrical test stimuli (at t = 0) before, during and after conditioned pain modulation (CPM) in a patient receiving burst, tonic and sham spinal cord stimulation paradigms*.*

The evoked response, in both regions of interest, elicited by the test stimulus was averaged across the 22 test stimuli per CPM condition. In both regions of interest, the highest amplitude of the evoked response was observed under sham SCS, followed by tonic SCS (Fig. 1). The lowest amplitude was observed under burst SCS, moreover under burst SCS no obvious components were observed beyond 250 ms, whereas later components were observed under tonic and sham SCS. The latest components were observed under sham SCS, extending until 1000 ms post-stimulus. Therefore, the area under the curve was calculated from 40 – 1000 ms post-stimulus (Table 2).

**Table 2:** Area under the curve of the evoked response in the foot area of the left primary somatosensory cortex and anterior cingulate cortex.

**ROI**	**SCS paradigm**	**AUC**			**Percentage change AUC**	
		Before CPM	During CPM	After CPM	Before vs. during CPM	Before vs. after CPM
**LeftS1**	Burst	2.2	2.2	2	2 %	−6 %
	Tonic	3.3	2.4	2.7	−27 %	−16 %
	Sham	6.7	4	4.7	−40 %	−30 %
**ACC**	Burst	2.4	2	2.5	−19 %	4 %
	Tonic	6.7	3.1	7.7	−54 %	15 %
	Sham	10.2	2.9	4.1	−72 %	−60 %

*AUC is calculated from 40 − 1000 ms after a painful stimulus before, during andafter CPM in a patient receiving burst, tonic and sham SCS paradigms*.

*ACC = anterior cingulate cortex; AUC = area under the curve; CPM = conditionedpain modulation; ROI = region of interest; SCS = spinal cord stimulation;S1 = primary somatosensory cortex*.

In the left S1, the evoked response was unchanged during CPM under burst SCS. In contrast, under tonic SCS the amplitudes of components at 400 and 600 ms were reduced during CPM. Under sham SCS all components of the evoked response were reduced during CPM.

In the anterior cingulate cortex (ACC) all components of the evoked response were largely reduced during CPM under burst, tonic and sham SCS.

## 4 Discussion

We evaluated whether the cortical evoked response could be used to evaluate the effect of conditioned pain modulation (CPM) in a chronic pain patient treated with burst, tonic and sham spinal cord stimulation (SCS) paradigms, with each paradigm having distinct effects on the patient’s chronic pain. Under all SCS paradigms, we observed a reduction in the amplitude of the evoked response elicited by the test stimulus during CPM, although the patient reported no reduction in the test stimulus pain rating. These findings suggest that evaluating changes in cortical activity during CPM, provides additional insights into CPM effects beyond self-reported pain ratings. By examining the cortical responses, we assess the net effect of both spinal and supraspinal mechanisms by SCS and CPM, making it challenging to definitively distinguish between their contributions.

### 4.1 Effects of SCS on the cortical evoked response

Comparing the before CPM condition across the different SCS paradigms allowed us to evaluate the effects of SCS on the evoked response elicited by painful stimuli. We observed a lower amplitude of the evoked response in the left S1 and ACC under tonic and burst SCS compared to sham SCS, likely due to spinal and supraspinal mechanisms that inhibit nociceptive input. The left S1 is primarily linked to the lateral pain pathway and associated with sensory aspect, while the ACC is linked to the medial pain pathway and associated with the suffering aspect (De Ridder et al., 2022). Burst SCS is thought to affect both pathways, whereas tonic SCS is thought to mainly affect the lateral pain pathway (De Ridder and Vanneste, 2016). This aligns with our findings: burst SCS strongly reduced left S1 and ACC activity, while tonic SCS strongly reduced left S1 activity and moderately reduced ACC activity compared to sham SCS.

Niso et al. (Niso et al., 2021) suggests that burst SCS reduces the cortical capacity to attend to somatosensory stimuli, resulting in a lower amplitude of evoked responses. SCS may have impaired the cortical capacity to attend to the painful test stimuli. Impaired cortical capacity to attend to stimuli under tonic and burst SCS may be linked to modulation of the salience network (De Ridder and Vanneste, 2016). The salience network overlaps with the medial pain pathway (De Ridder et al., 2022). Since burst SCS is thought to affect this pathway, it might explain its stronger inhibitory effect compared to tonic SCS.

Using painful transcutaneous electrical stimuli, we aimed to stimulate the nociceptive Aδ-fibres, however Aβ-fibre activation occurred as well (La Cesa et al., 2018). Previous studies have shown that tonic SCS inhibits the early and late components of the evoked response elicited by non-painful A β-fibre stimulation (Reinders et al., 2024). The effects of burst SCS on the evoked response elicited by A β-fibre stimulation are less researched, but burst SCS likely also inhibits the late components (Niso et al., 2021). Bocci et al. (Bocci et al., 2018), using laser-evoked potentials (LEPs) that specifically activate Aδ-fibres, reported strong inhibition by burst SCS and a weaker effect by tonic SCS (200–270 ms latency) in line with our results.

### 4.2 Effects of CPM on the cortical evoked response

Comparing the before CPM condition with the during CPM condition (parallel paradigm) allowed us to evaluate the effects of CPM on the evoked response. CPM inhibited the evoked response in the left S1 under sham and tonic SCS and in the ACC under all SCS paradigms. The observed inhibition may stem from involvement of the spino-bulbo-spinal loop and from modulation of cortical structures related to the descending pain pathway. Additionally, the conditioning stimulus may have impaired cortical capacity to attend to the test stimulus.

In the during CPM condition, the conditioning stimulus may have caused distraction from the test stimuli, despite instructing the patient to attend to the test stimuli and her correctly counting them. Comparing the before CPM condition with the after CPM condition (sequential paradigm) allowed us to evaluate the effects of CPM on the evoked response, while minimizing potential influence of distraction from the test stimuli by the conditioning stimulus (Ramaswamy and Wodehouse, 2021). After CPM, the amplitude of the evoked response in the S1 was inhibited under all SCS paradigms, while in the ACC, inhibition remained only under sham SCS. Therefore, possibly the observed inhibited ACC activity during CPM under tonic and burst SCS is (partly) due to distraction.

### 4.3 Effects of SCS on CPM

If active SCS already reduces the cortical capacity to attend to painful test stimuli, there is little room for the conditioning stimulus to further reduce cortical capacity. Following this theory, the cortical capacity to attend to stimuli was most strongly reduced under burst SCS, as the lowest amplitudes of the evoked responses across all CPM conditions were observed. Moreover, during CPM the inhibition of the evoked response was minimal under burst SCS. These findings can be explained by burst SCS exerting a greater influence on the salience network through its effect on the medial pain pathway.

These findings, observed in one subject, should be validated and further investigated in a larger scale study to enhance our understanding of the mechanisms of various SCS paradigms.

## 5 Conclusion

In this case study we were able to evaluate conditioned pain modulation (CPM) using cortical evoked responses in a chronic pain patient treated with burst, tonic and sham spinal cord stimulation (SCS). While CPM reduced the amplitude of the evoked response elicited by the test stimulus, the patient reported pain ratings were unaffected. This discrepancy suggests that the use of neurophysiological measures, such as the evoked response, may provide additional insights into CPM effects beyond self-reported pain ratings. Both tonic and burst SCS might affect the salience network, resulting in reduced cortical capacity to attend to painful stimuli. The reduction in cortical capacity to attend to stimuli was the strongest under burst SCS. This case study indicates that neurophysiological measures can serve as a valuable tool in understanding the effect of SCS on the pain pathways, our findings should be validated and further investigated in a larger scale study.

## 6 Authorship statements

CV was involved in the study design and acquisition of the data. LR conducted the data analysis with input from CV. CV and LR collaboratively interpreted the results. LR wrote the initial manuscript, and both LR and CV critically revised and finalized the manuscript.

## Declaration of Competing Interest

The authors declare that they have no known competing financial interests or personal relationships that could have appeared to influence the work reported in this paper.
